# Tackling the challenge of chronicity through healthcare integration in Gipuzkoa Oeste Health Region/El reto de la cronicidad a través de la integración asistencial en la Comarca Guipúzcoa Oeste

**Published:** 2012-05-29

**Authors:** Marisa Merino Hernandez, Elena Baylin Zaldua, Mariluz Jauregui Garcia, Javier Basterrechea Peña, Amaya Hernando Uzkudun

**Affiliations:** Gipuzcoa Health Region, San Sebastián, Spain; Goierri-Alto Urola Integrated Health Organisation, Zumárraga, Spain; Goierri-Alto Urola Integrated Health Organisation, Zumárraga, Spain; Gipuzcoa Health Region, San Sebastián, Spain; Goierri-Alto Urola Integrated Health Organisation, Zumárraga, Spain

**Keywords:** chronicity, clinical integration, health care, cronicidad, integración clínica, sanidad

## Introduction

Ageing, a constant rise in demand for healthcare, rapid and continuous technological and pharmacological innovation and a growing pressure on financial resources are some of the reasons for needing renewal in our health system. Chronic patients account for 80% of primary care consultations, 60% of hospital admissions and 70% of health expenditure, and their number and complexity is ever increasing. The provision of services is fragmented and chronic patients have special requirements in this respect, since the care they need tends to involves the participation of several centres, a range of specialists, many different drugs, etc.

Given all this and following the strategy of the Department of Health of the Government of the Basque Country [1], it was suggested that new organisational healthcare models were required, redefining care pathways to improve coordination and avoiding inefficiencies in the health service.

## Description of the initiative

In this context, we decided to establish an integrated healthcare model for chronic patients in the Gipuzkoa Mendebalde health region that would promote continuity of care, enhance relationships between the healthcare and social sectors and be sustainable. Accordingly, the key factors would include: efficiency, continuity, safety, quality, innovation and research. The following organisations in the Basque Health Service (Osakidetza) participated in the project: Gipuzkoa Oeste primary health region; the public hospitals of Zumarraga, Alto Deba, and Mendaro; and Asunción Hospital, a private provider.

The project was developed on the basis of methodologies, such as “project management”, “process management”, “techniques to promote creativity and innovation”, “nominal group techniques”, “learning and development of competences” and “action research approaches”. It was launched in January 2010 and is ongoing. The Table 1 indicates the most important milestones in its development.

At the time of writing, a clinical integration model has been introduced, the conceptual framework of which is founded on the Chronic Care Model [2, 3] and the WHO Innovative Care for Chronic conditions (ICCC) framework [4–6]. It is patient-centred and based on clinical leadership and the involvement of health professionals from both levels of care. The population is stratified following the model of the risk pyramid and, for each level, the most appropriate type of intervention is indicated. The strategic lines defined and the projects planned for each are mentioned below.

In the Table 2, we list the main interventions to be carried out for each level in the risk pyramid.

### Integration of care processes

In collaboration with the hospitals of Zumarraga and Alto Deba, an emergency care process has been designed and it has been adopted in half of the health region.

### Collaboration between healthcare and social services

- Introduction of the use of oral anticoagulation therapy in residential homes for the elderly
- Inter-institutional care protocol for patients with hip fractures
- Inter-institutional care protocol for victims of domestic violence
- “*Etxean ondo*” Project for home care of elderly individuals

### Empowering patients

- Clinical trial on the effectiveness of spirometry as a motivational instrument for smoking cessation in patients with chronic bronchitis
- Group education
- Expert patient project

### New healthcare model

The following pilots are being run in several primary care units:

- Evaluations of new care roles for nurses
- Nursing triage and management of mild self-limiting conditions in paediatrics

### Innovation

Some health centres have managed to become paper-free and this pilot is being extended.

### Evaluation

The new project, studies and interventions are introduced as pilots, their usefulness being evaluated before they are adopted in other areas. We have also collaborated with Osakidetza central management services in the development and introduction of an evaluation framework for healthcare integration pilots, which will improve the assessment of the various projects underway as well as enabling us to make comparisons with other organisations [27].

## Discussion

We have defined a new model of integrated healthcare model in the Gipuzkoa Oeste health region that is patient-focused and based on clinical leadership and the involvement of health professionals.

General strategic lines and specific projects in each area have been developed. We expect that coordinated and simultaneous interventions in various different areas will significantly improve patient continuity of care, health outcomes, perceived quality and the satisfaction of health professionals and patients.

In particular, the establishment of indicators for evaluating both the model and specific projects will enable us to assess the interventions and, in turn, achieve continuous improvement in patient care.

## Poster abstract Spanish

## Introducción

El envejecimiento, el aumento constante de la demanda, la rápida y continuada innovación tecnológica y farmacológica, y una necesidad creciente de recursos económicos, son algunos de los motivos que nos conducen a la necesidad de renovar nuestro sistema sanitario. Los pacientes crónicos suponen el 80% de las consultas de atención primaria, el 60% de los ingresos hospitalarios y el 70% del gasto sanitario, estando en continuo aumento, tanto en número como en complejidad. La prestación de servicios está fragmentada y los pacientes crónicos tienen una especial necesidad en este sentido, ya que con frecuencia deben de integrar la atención recibida en distintos centros, diversos especialistas, distintos fármacos etc.

Considerando lo anterior y siguiendo las líneas estratégicas del Departamento de Sanidad del Gobierno Vasco [1], se planteó la necesidad de nuevos modelos de organización de la atención redefiniendo circuitos asistenciales que mejoraran la coordinación, evitando ineficiencias del sistema sanitario.

## Descripción de la experiencia

En este contexto, se planteó la creación de un Modelo de Asistencia Sanitaria Integrada al paciente crónico en la Comarca Gipuzkoa Oeste que favoreciera la continuidad asistencial, la relación con el entorno sociosanitario y que fuera sostenible. Por tanto, los factores clave debían ser eficiencia, continuidad, seguridad, calidad, innovación e investigación.

El proyecto está conformado por distintas organizaciones de servicios de Osakidetza (Servicio Vasco de SaludComarca de Atención Primaria Gipuzkoa Oeste, los hospitales de Zumárraga, Alto Deba, y Mendaro, y la Clínica de la Asunción, que es un hospital de carácter privado.

El proyecto se ha desarrollado en base a metodologías tales como “gestión de proyectos”, “gestión de procesos”, “técnicas para fomentar la creatividad y la innovación”, “grupos nominales”, ”aprendizaje y desarrollo de competencias” y “estudios de investigación-acción”.

El proyecto arrancó en enero del año 2010 y a día de hoy sigue vivo. La Tabla 3 presenta los hitos más importantes en el desarrollo del mismo.

A día de hoy, se ha implantado un modelo de integración clínica cuyo marco conceptual parte del modelo de enfermedades crónicas CCM (Chronic Care Model) [2–3] y del proyecto ICCC (Innovative Care for Chronic conditions) de la OMS [4–6].

Está centrado en el usuario y se basa en el liderazgo clínico y la implicación de profesionales de ambos niveles. La población se estratifica según el modelo de la pirámide de riesgo, indicándose el tipo de actuación más adecuada en cada caso. Las líneas estratégicas definidas y los proyectos planteados se mencionan a continuación.

En la Tabla 4 se exponen las principales intervenciones llevadas a cabo en cada uno de los niveles de la pirámide.

### Integración de procesos de atención

Se ha diseñado el proceso de atención urgente e implantado en la mitad de la comarca, junto con los hospitales de Zumarraga y Alto Deba.

### Colaboración socio-sanitaria

- Implantación de la anticoagulación oral en residencias de ancianos
- Protocolo de atención interinstitucional a pacientes con fractura de cadera
- Protocolo interinstitucional de atención a víctimas de violencia de género
- Proyecto “Etxean ondo” de asistencia domiciliaria

### Empoderamiento del paciente

- Ensayo clínico sobre la efectividad de la espirometría como instrumento motivacional para dejar de fumar en pacientes con bronquitis crónica
- Educación grupal
- Paciente activo

### Nuevo modelo asistencial

En varias unidades de atención primaria se están pilotando:

- Experiencias para valorar nuevos roles asistenciales de enfermería.
- Triaje de enfermería y atención a procesos leves autolimitados en pediatría

### Innovación

Se ha conseguido que varios centros de salud trabajen sin papeles y se va extendiendo la experiencia.

### Evaluación

Los nuevos proyectos, estudios o acciones se ponen en práctica como pilotos, evaluando su utilidad, previo a la extensión a otras zonas. También se ha colaborado con los servicios centrales de gestión de Osakidetza en el desarrollo e implantación de un marco evaluativo para las experiencias de integración asistencial, que permitirá valorar los distintos proyectos que están en marcha y la comparación con otras organizaciones [27].

## Discusión

Se ha definido un nuevo modelo de atención sanitaria integrada en la Comarca Gipuzkoa Oeste centrado en el paciente y basado en el liderazgo clínico y la implicación de los profesionales.

Se han planteado las líneas estratégicas generales y los proyectos concretos en cada una de ellas. Se espera que la actuación coordinada y simultánea en diversas áreas mejore de forma significativa la continuidad asistencial de los pacientes, los resultados en salud, la calidad percibida y la satisfacción de los profesionales y pacientes.

El establecimiento de indicadores para evaluar tanto el modelo como los proyectos concretos permitirá valorar las experiencias y abordar la mejora continua de la atención a los pacientes.

## Figures and Tables

**Table 1.** Milestones

Date	Milestones
01/2010	Design of the project
01/2010	Communication plan for heads of primary care (PC) centres and regional hospitals
01-03/2010	Literature review [1–26]
04/2010	Establishment of a working group with primary care professionals
06/2010	Formulation of a proposal by the primary care group
09/2010	Preparation of research projects
10/2010	Establishment of mixed PC-specialised care (SC) working groups involving the hospitals
12/2010	Collaboration in the definition of an evaluation framework
12/2010-01/2011	Descriptive study of complex chronic patients in the health region
01/2011	Creation of a specific Google site to support networking
02/2011	Nominal group sessions run with health professionals and patients
02/2011	Meeting held on creativity applied to chronicity
04/2011	Common strategic lines defined and pilots proposed

**Table 2.** Interventions as a function of the risk

**Level 3: Management of complex cases: High degree of comorbidity and high use of resources**
• Integrated care for patients with multiple diseases and/or on multiple medications
• Introduction of the role of referral internist and consultant
• Pilot: competences for advanced nurse case managers
• Pilot: competences for liaison nurses
• Patient stratification
**Level 2: Management of diseases: Intermediate degree of comorbidity and high use of resources**
• Design and introduction of integrated care pathways for chronic bronchitis, heart failure, diabetes, palliative care, and atrial fibrillation
• Clinical trial on chronic bronchitis
• Research study: Identification of pre-diabetic patients
• Multicentre study to assess the effectiveness of telemedicine in quality assurance programmes for spirometry tests
• Establishments of priority pathways
**Level 1: Support for self-management: Chronic patients with good control of their disease**
• Design and carry out pilots on new roles for nurses
• Research study: group sessions for initiation of insulinisation in patients with type 2 diabetes
• Expert patient project
• Patient stratification
**All levels: Improvement in prevention and promotion**
• Research project: “*Prescribe vida saludable*” (for promoting healthy lifestyles)
• Population screening for colon cancer
• Management of cardiovascular risk: group education
• Diagnostic studies to assess the health status of patients over 75 years of age
• Promotion of physical activity in the elderly and children in collaboration with the Department of Public Health: “*Tipi-tapa*” and “*Amarauna*” projects
• Smoking intervention programmes

**Tabla 3.** Hitos

FECHA	HITOS
01/2010	Diseño del proyecto
01/2010	Plan de comunicación a JUAPs y hospitales comarcales
01-03/2010	Revisión de bibliografía [1–26]
04/2010	Creación de grupo de trabajo con profesionales de atención primaria
06/2010	Elaboración de propuesta por parte del grupo de atención primaria
09/2010	Elaboración de proyectos de investigación
10/2010	Creación de grupos de trabajo mixtos AP-AE con los hospitales
12/2010	Colaboración en la definición de un marco evaluativo
12/2010-01/2011	Estudio descriptivo de los pacientes crónicos complejos de la comarca
01/2011	Creación de un Google site específico que apoye el trabajo en red
02/2011	Realización de grupos nominales con profesionales y pacientes
02/2011	Jornada sobre creatividad aplicada a la cronicidad
04/2011	Elaboración de líneas estratégicas comunes y propuestas de experiencias piloto

**Tabla 4.** Intervenciones según la pirámide de riesgo

**Nivel 3: Gestión de casos complejos: Mucha comorbilidad y alto uso de recursos**
Atención integral al paciente pluripatológico y/o polimedicado
Establecimiento de médico internista de referencia y consultor
Pilotaje competencias enfermera gestora de casos avanzada
Pilotaje competencias enfermera de enlace
Estratificación de pacientes
**Nivel 2: Gestión de patologías: Morbilidad intermedia y alto uso de recursos**
Diseño e implantación de rutas asistenciales integradas: bronquitis crónica, insuficiencia cardiaca, diabetes, cuidados paliativos y fibrilación auricular.
Ensayo clínico bronquitis crónica
Estudio de investigación identificación de pre-diabético
Estudio multicéntrico para analizar la efectividad de la telemedicina en programas de aseguramiento de la calidad de las espirometrías
Establecimiento de circuitos prioritarios
**Nivel 1: Apoyo a la autogestión: Pacientes enfermos crónicos con buen control de su enfermedad**
Diseño y pilotaje de nuevos roles en enfermería
Estudio investigación iniciación grupal insulinización en diabetes tipo 2
Paciente activo
Estratificación de pacientes
**Todos los niveles: Mejora de la prevención y promoción**
Proyecto investigación: Prescribe vida saludable (para la promoción de estilos de vida saludables)
Cribaje poblacional cáncer de colon
Manejo del riesgo cardiovascular: educación grupal
Estudio diagnóstico situacional de pacientes mayores de 75 años
Fomento de la actividad física en ancianos y niños junto con Salud Pública: proyectos Tipi-tapa y Amarauna
Programa de intervención en tabaquismo
